# Social Inequalities and Health among Older Immigrant Women in the Nordic Countries: An Integrative Review

**DOI:** 10.1177/23779608221084962

**Published:** 2022-03-14

**Authors:** Jonas Debesay, Line Nortvedt, Birgitta Langhammer

**Affiliations:** 1 Department of Nursing and Health Promotion, OsloMet – 60499 Oslo Metropolitan University, Oslo, Norway; 2 Department of Physical Therapy, OsloMet – 60499 Oslo Metropolitan University, Oslo, Norway

**Keywords:** ethnic minority, aging, female, equitable, healthcare

## Abstract

**Introduction:**

The Nordic countries have a surprisingly strong relative socioeconomic health inequality. Immigrants seem to be disproportionately affected due to their social economic position in the host countries. Healthcare professionals, including nurses, have a professional obligation to adhere to fairness and social equity in healthcare. The aim of this review was to identify and synthesize research on health status and the impact of social inequalities in older immigrant women in the Nordic countries.

**Methods:**

We conducted an integrative review guided by the Whittemore and Knafl integrative review method. We searched multiple research databases using the keywords immigrant, older, women, socioeconomic inequality, health inequality, and Nordic countries. The results were limited to research published between 1990 and 2021. The retrieved articles were screened and assessed by two independent reviewers.

**Results:**

Based on the few studies on older immigrant women in the Nordic countries, the review findings indicate that they fare worse in many health indicators compared to immigrant men and the majority population. These differences are related to various health issues, such as anxiety, depression, diabetes, multimorbidity, sedentary lifestyle, and quality of life. Lower participation in cancer screening programs is also a distinctive feature among immigrant women, which could be related to the immigrant women's help-seeking behavior. Transnational family obligations and responsibilities locally leave little room for prioritizing self-care, but differing views of health conditions might also contribute to avoidance of healthcare services.

**Conclusion:**

This integrative review shows that there is a paucity of studies on the impact of social inequalities on the health status of older immigrant women in the Nordic countries. There is a need for not only research focused on the experiences of health status and inequality but also larger studies mapping the connection between older immigrant women's economic and health status and access to healthcare services.

## Introduction

Social inequality, measured as education level, employment/occupation, and income, is one of the biggest challenges for public health worldwide (Mackenbach et al., 2008, 2016). In Denmark, Finland, Norway, Sweden, and Iceland, there exists an “unexpectedly strong relative socioeconomic health inequality” (Huijts & Eikemo, 2009, p. 452), despite the universal provision of goods and services, which is a distinctive feature of the Nordic countries’ welfare models (Esping-Andersen, 1990). This systematic difference indicates that those with higher education and income in general have better health (Grøholt et al., 2019). Cardiovascular disease, lung cancer, and chronic lung disease account for almost 60% of the difference in mortality before the age of 67 years between those with low and high education (Elstad et al., 2009). Differences in lifestyle habits and prevalence of the classic risk factors may explain a significant portion of the social differences in heart disease mortality in Norway (Strand & Tverdal, 2004), Sweden (Kilander et al., 2001), and other countries (Beaglehole & Magnus, 2002).

The immigrant population has become a sizable proportion of the general population in the Nordic countries, varying from 7.1% in Finland to 12.3% in Denmark, 16.2% in Norway, 17.9% in Iceland, and 19.5% in Sweden (Eurostat, 2021). Immigrants, in contrast to the rest of the population, have increased health problems and encounter problems accessing health services due to lower socioeconomic levels (Ahmed et al., 2017; Bas-Sarmiento et al., 2017; Semedo et al., 2019). Similarly, their overall consumption of different forms of healthcare services is, in many respects, lower than that of the general population (Debesay et. al., 2019). Many might be at a higher risk of illness and health problems due to their age and socioeconomic disadvantages (Beard et al., 2016). As the older populations are increasing in all the Nordic countries (Jørgensen et al., 2019), a significant increase among older immigrants is expected (Carling et al., 2015; Thomas & Syse, 2021; Tønnessen & Syse, 2021).

Furthermore, there are gender differences in social inequalities in health. For example, smoking is more common among men, while obesity is more common among women (Mackenbach et al., 2008). Compared to other high-income countries, men in the Nordic countries have the highest life expectancy (Popham et al., 2013). Immigrant women are also known to have a higher post-migration risk of health problems compared to men (Lopez-Gonzalez et al., 2005; Vrålstad & Wiggen, 2017). However, studies also indicate a so called “healthy immigrant effect” showing that immigrants might have better health (Biddle et al., 2007), equal access to some healthcare services (Ohlsson et al., 2016), or even higher life expectancy compared to the majority population in the host country (Mehta et al., 2016; Vang et al., 2017).

As social inequality in health cuts across the general population, the most efficient strategy to alleviate such inequalities is to address the population most at risk, especially immigrants. This strategy would, presumably, give a significant boost to public health in general. However, there is a need for more knowledge about social differences in the use of healthcare and what might contribute to or counteract such differences (Dahl et al., 2014).

Healthcare professionals, including nurses, have a professional obligation to adhere to fairness and social equity in healthcare (Massey & Durrheim, 2007). Therefore, there is an urgent need for more knowledge about social inequalities and health in the immigrant population, especially of those most disadvantaged, such as older immigrant women from Asia, Africa, and South America (Huijts & Eikemo, 2009). The aim of this integrative review was to identify and synthesize research on health status and the impact of social inequalities in older immigrant women in the Nordic countries. We sought to examine research addressing health status among older immigrant women and to what extent social inequalities influence their health.

## Methods

This integrative review is a synthesis of qualitative and quantitative studies. An integrative review approach is suitable when the research aim is broad, and because this approach enables a comprehensive synthesis across research methods. This way, we have been able to capture the context of older women as well as their subjective experiences related to social inequalities and health (Hardin et al., 2021; Whittemore & Knafl, 2005).

### Data Sources and Search Strategy

The review aimed to identify peer-reviewed primary studies concerning the health status among older immigrant women from Asia, Africa, and South America (≥50 years) in the Nordic countries, and whether or how social inequalities influence health, and which factors are most influential, such as (family) income, educational level, occupation/employment, lifestyle. Papers from 1990 to 2021 were included in the review. We chose to include studies of women older than 50 years of age, in contrast to those older than 60 or 65 years, as is often the case in the immigrants’ host countries. Many immigrants describe themselves as “older” in their 50 s, which is also in accordance with the World Health Organisation's (WHO) recommendations for people from low-income countries (Emami & Ekman, 1998; Molsa et al., 2014). Although we include the different uses of the term “health” in the presented literature, we apply the WHO definition of health as a basis: “Health is a state of complete physical, mental and social well-being and not merely the absence of disease or infirmity.” (WHO, 2022).

Eight databases were searched in June 2020: MEDLINE (OVID, 1946–), Embase (OVID, 1980–), AMED (OVID, Allied and Complementary Medicine, 1985–), APA PsycINFO (OVID, 1986–), CINAHL (EBSCOhost), SocINDEX (EBSCOhost), Web of Science (Clarivate Analytics, Indexes: SCI-EXPANDED, SSCI, A&HCI, 1987–, ESCI 2015–), and SweMed + (Karolinska Institutet, 1990–2019). The searches were updated in June 2021 (for the period January 2020–June 2021). In addition, a search in the Norwegian library resource Oria was conducted due to the focus on the Nordic countries. The search strategy included elements from the patient/population, intervention, comparison, and outcomes model (PICO) (Table 1).

**Table 1.** PICO Model for the Search Strategy.

Population	Intervention	Comparison	Outcomes
Women ≥50 years	Not applicable	Income	Not specified
Immigrant women		Education	
Health: physical, psychological		Employment	
Nordic countries		Literacy	
		Lifestyle	

The elements above were searched with relevant terms from the different databases as controlled vocabulary and as free text (Table 2). Due to a lack of resources for translation, references published in languages other than English or the Nordic languages were excluded during the screening process. The limiter was publication date (1990–2020/2021), and in SweMed + , the results were also limited to peer-reviewed journals. The reference management software EndNote (version X9) was used to find and remove duplicate references, and for the screening process. The systematic and peer-reviewed literature search was conducted by the Literature Search Expert Group at OsloMet.

**Table 2.** Search words.

(female* or woman* or women*) (immigra* or emigra* or ethnic* or multi-cultur* or cross-cultur* or crosscultur* or native* or nativity)
AND
MH “Immigrants”) OR (MH “Emigration and Immigration”) OR (MH “Minority Groups”) OR (MH “Ethnic Groups”) OR (MH “Cultural Diversity”) OR (MH “Transcultural Care”) Racial groups or minority groups innvandr* flerkultur* or multikultur* or krysskultur*
AND
(MH “Scandinavia”) OR (MH “Denmark”) OR (MH “Finland”) OR (MH “Norway”) OR (MH “Sweden”) OR (MH “Iceland”) (norw* or swed* or danish or Denmark or danes or Finland or finns or finnish or Iceland* or Scandinavia* or nordic)
AND
(MH “Socioeconomic Factors”) OR (MH “Social Class”) OR (MH “Economic Status”) OR (MH “Illiteracy”) OR (MH “Poverty”) OR (MH “Literacy”) OR (MH “Educational Status”) OR (MH “Employment”) OR (MH “Employment Status”) OR (MH “Unemployment”) OR (MH “Income”) OR (MH “Healthcare Disparities”) OR (MH “Social Determinants of Health”) (social* or socio* or inequal* or unequal* or equal* or “low income*” or poor or poverty or disparit* or educat* or uneducate* or literacy or literate or illitera* or employ* or unemploy* or SES) economic status or income sosial* or sosio* or utdann* or utbild* fattig* or ulikhet* or jämlikhet* or olikhet*
AND
(MH “Health”) OR (MH “Health and Disease”) OR (MH “Disease”) OR (MH “Health Status Disparities”) OR (MH “Health Status”) OR (MH “Social Determinants of Health”) OR (MH “Mental Health”) OR (MH “Psychological Well-Being”) (health* or unhealth* or well* or sick* or illness* or disease*) helse* or sykdom* or sjukdom* or uhelse

### Screening Process

All steps in the systematic study are reported by using the Preferred Reporting Items for Systematic reviews (PRISMA) statement and checklist (Moher et al., 2009; Tricco et al., 2018). Data sources were screened, and full texts were reviewed by at least two authors at all stages, using the Covidence literature screening software which allows for a streamlined review process and blinded double screening in all study phases (Covidence, 2021).

**Figure 1.**
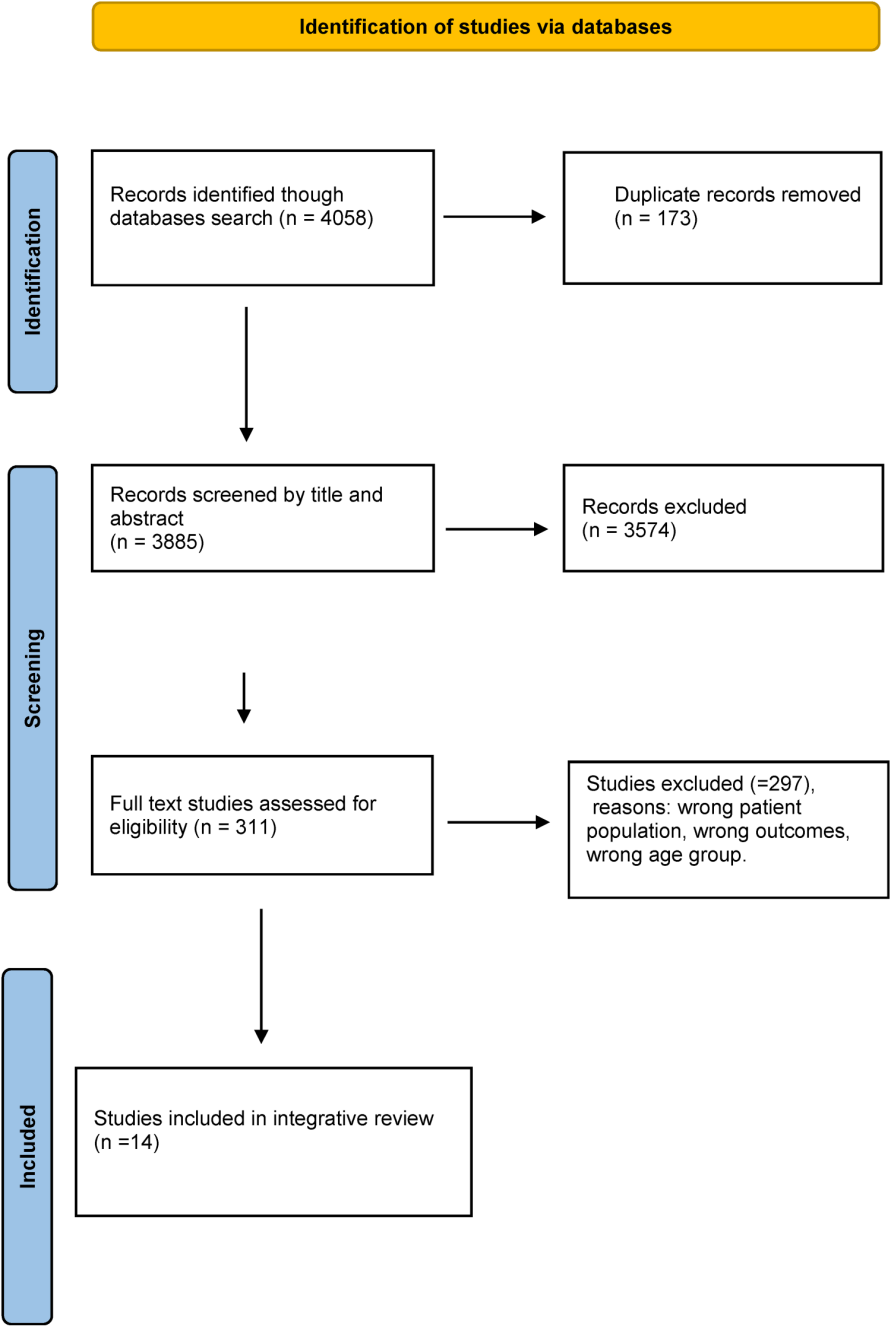
PRISMA flow diagram for the search social inequalities and health among older immigrant women.

The review process included the following steps: 1) screening titles, 2) reading abstracts, 3) reading full-text articles (see the PRISMA flow chart in Figure 1), and 4) assessing the quality of the selected studies.

The title and abstract selection was based on the published studies’ relevance to the topic, our exclusion criteria, and whether the studies provided information distinguishing among immigrants’ country of birth or lacked sub-analyses of older immigrants or/and women.

We used the MMAT, a quality assessment tool designed to critically evaluate quantitative, qualitative, and mixed methods studies that are included in systematic mixed studies. We employed the MMAT as a checklist for simultaneous evaluation of the papers included in this integrative review study (Hong et al., 2018). The systematic review is registered at the Joanna Briggs Institute and JBI Systematic review and PROSPERO CRD42020160995, 200720.

## Results

### Characteristics of the Studies

The search for empirical studies on health status and social inequalities and immigrant women in the Nordic countries resulted in 10 quantitative (cross-sectional or cohort) and 4 qualitative (one focus group and three one-to-one interviews) primary studies (Tables 2 and 3). All 14 studies were of good quality and met the MMAT's assessment criteria (Table 4). The studies originated from Sweden (7), Denmark (3), Norway (3), and Finland (1) (Table 5).

**Table 3.** Description of the Qualitative Papers Included in the Review (n = 4).

Author	Title	Year	Study design	Data Collection Methods	Target sample Sample size Mean age, Sampling Method	Results Elderly women
Emami, A.; Ekman, S.	Living in a foreign country in old age: life in Sweden as experienced by elderly Iranian immigrants	1998	Descriptive qualitative study Invited convenient sample from a register Stockholm City Data service	In-depth interviews Tape recorded, phenomenological-hermeneutics methodology	123 invited. 30 elderly Iranians living in Stockholm were interviewed (18 men and 12 women).	>75 years of age: Gratitude for help and support/ welfare services feel lonely. Only social activity: Spend time with children/other relatives. Hobbies: Daily walking. Considered themselves to be quite healthy, but also described diffuse pain, abdominal pain, headaches and sleeplessness.
Emami, A.; Tishelman, C.	Reflections on cancer in the context of women's health: focus group discussions with Iranian immigrant women in Sweden	2004	Low-income brackets in Sweden	9 Focus group interviews	45 females from 3 age groups; 25–35, 36–45 and 55–70	Menopause seen as positive. The notion of self and body as a whole; “beauty comes from inside”. Acceptance of social roles; women's fate was to be subordinate men. Health as a continuum in life, diseases as part of a normal life.
Kessing, L. L.; Norredam, M.; Kvernrod, A. B.; Mygind, A.; Kristiansen, M.	Contextualising migrants’ health behaviour - a qualitative study of transnational ties and their implications for participation in mammography screening	2013	Convenient sample qualitative interviews	Semistructured interviews; 8 individual interviews and 6 focus group interviews.	N = 29 women, aged 50–69 years. Majority: only primary school education.	Barriers for not attending the screening programme: Inability to read the invitation, lack of transport/emotional or social support, life stressors and competing priorities, as multiple diseases and maintaining relationships with transnational relatives.
Kristiansen, M.; Lue-Kessing, L.; Mygind, A.; Razum, O.; Norredam, M.	Migration from low- to high-risk countries: a qualitative study of perceived risk of breast cancer and the influence on participation in mammography screening among migrant women in Denmark	2014	Phenomenological study.	13 semi-structured interviews (8 individual and 6 group interviews)	29 females Age: 50–69 years	*Educational attainment and employment rates were low* among the participants, and few had participated in the mammography screening programe. Breast cancer was perceived to be caused by multiple factors, including genetics, health behaviour, stress, fertility and breastfeeding. perceived their risk of developing breast cancer to increase with length of stay in Denmark.

**Table 4.** Description of the Quantitative Papers Included in the Review (n = 10).

Author	Title	Study aim	Year	Study design	Data Collection Methods	Target sample Sample size Mean age, Sampling Method	Elderly women
Abuelmagd, W,HakonsenH,Mahmood KQ, Taghizadeh N, Toverud E L.	Living with Diabetes: Personal Interviews with Pakistani Women in Norway	To assess how Pakistani women in Norway live with their type 2 diabetes (T2D) and how they respond to lifestyle and medical information.	2018	Cohort	A questionnaire used in face-to-face personal interviews descriptive statistics	n = 120 Pakistani women 29−80 years of age mean 55.7 years.	> 50 years of age (n = 71 poor T2D control among in terms of lifestyle habits, comorbidities, and drug use. Low literacy and cultural factors seem to challenge adherence
Diaz E, Kumar B N, Gimeno-Feliu L A, Calderon-Larranaga A, Poblador-Pou B, Prados-Torres A.	Multimorbidity among registered immigrants in Norway: the role of reason for migration and length of stay	To explore the association between multimorbidity and reason for migration. - to compare the impact of length of stay in Norway and other socio-demographic variables	2015	A register-based study: National Population Register and the Norwegian Health Economics Administration database (HELFO).	Comparisons of sociodemographic variables and multimorbidity across the four immigrant groups Persons’ chi-square test and anova as appropriate models of binary logistic regression analyses	A total of 67,398 refugees, 66,942 labour immigrants, 101,276 family reunification immigrants and 16,379 education immigrants, Mean age 29–36 years >65 years 1924 family reunification, 134 labour immigrants, 2292 refugees	Multimorbidity was significantly lower among labour and education immigrants and higher among refugees than family reunification immigrants. For all groups, multimorbidity doubled after 5 years of living in Norway.
Koochek, A.; Johansson, S. E.; Kocturk, T. O.; Sundquist, J.; Sundquist, K.	Physical activity and body mass index in elderly Iranians in Sweden: a population-based study	To analyze whether elderly Iranians in Sweden have a higher mean body mass index (BMI) and are less physically active than elderly Swedes	2008	Cross sectional	Linear regression and unconditional logistic regression	402 men and women (167 Iranian-born and 235 Swedish-born) aged 60–84 years	Iranian women had significantly higher BMI than the reference group after adjustment for age, education, and marital status. No difference in PA between groups
Koocheck A, Montazeri A, Johansson SE, Sundquist J	Health related quality of life and migration: a cross sectional study on elderly Iranians in Sweden	To examine the association between migration status and HRQL in a comparison of elderly Iranians in Iran, elderly Iranian immigrants in Sweden, and elderly Swedes in Sweden	2007	Cross sectional	Multiple linear regression	Iranians Iran = 298. Iranians in Sweden = 176, Swedish control group = 151	The HRQL of elderly Iranians in Sweden was more like that of their countrymen in Iran than that of Swedes, who reported a better HRQL than Iranians Increased time in Sweden – increased HRQoL in elderly Iranian women but not men
Kristiansen, M.; Thorsted, B. L.; Krasnik, A.; Von Euler-Chelpin, M.	Participation in mammography screening among migrants and non-migrants in Denmark	To explore: 1) the effects of determinants related to socioeconomic position, social support and use of healthcare services on participation in mammography screening 2) whether effects of determinants were consistent across migrant and non-migrant groups.	2012	Cohort from the first eight invitation rounds of the mammography screening programme in Copenhagen, Denmark (1991–2008)	Logistic regression was used to calculate odds ratios	Danish-born women n = 84,489, 74% were users of the organized mammography screening programme. women born in other-Western countries n = 5484 67% users, women born in non-Western countries n = 5891, users 61%	being 60–64 years old, non-Western women in this age-group were the least likely to participate in mammography screening. (36, 35 and 46% respectively)
Le, M., Hofvind. S., Tsuruda, K., Braaten, T. & Bhargava, S.	Lower attendance rates in BreastScreen Norway among immigrants across all levels of socio-demographic factors: A population-based study.	To identify the extent to which sociodemographic factors were associated with attendance among immigrant and non-immigrant women invited to the program during the period from 1	2019	Cohort	Poisson regression	885979 Women aged 50-69 (Age 25 to 67 included in the study)	53% of immigrants and 76% of non-immigrants attended mammographic screening after their first invitation. Factors associated with non-attendance were low income, living in Oslo, not being employed and being a recent immigrant
Lindstrom, M.; Sundquist, J.	Immigration and leisure-time physical inactivity: a population-based study	The association between immigrant status and the risk of having a sedentary leisure-time physical activity status	2001	A cross-sectional study	Interviewed by means of a postal questionnaire multivariate analysis	n = (3861–73) 3788 aged 20–80 years public health survey response rate = 71%	n = 982 elderly ladies 58years 244 68 years 249 78years 264 88 years 225 substantial ethnic differences in physical inactivity between immigrants and Swedes which could not be explained by confounders such as age and educational status
Molsa, M.; Punamaki, R. L.; Saarni, S. I.; Tiilikainen, M.; Kuittinen, S.; Honkasalo, M. L.	Mental and somatic health and pre- and post-migration factors among older Somali refugees in Finland	To investigate mental and somatic health among older (>50 years) refugees	2014	Somali-speaking interviewers Finnish speaking: paper version	ANOVA Kji^2^ statistics linear multiple stepwise regression	n = 128 Somali n = 128 matched controls Age 50-80	Somali group reported poorer current health and quality of life than their male counterparts, no gender differences were found in the Finnish group.
Steiner, K. H.; Johansson, S. E.; Sundquist, J.; Wandell, P. E.	Country of birth-specific and gender differences in prevalence of diabetes in Sweden	To investigate country- or region-specific prevalence of diabetes and gender differences in residents in Sweden based on their country of origin, using Swedish-born men and women as referent	2013	Cross sectional Self-Reported Anxiety, Sleeping Problems and Pain Among Turkish-Born Immigrants in Sweden	Differences between men and women were calculated by means of kji2 -tests using the age-standardized prevalence numbers	526 Turkish-born immigrants in Sweden were compared with 2,854 Swedish controls, all aged between 27 and 60 years.	Using Swedish women as control, Turkish-born women showed an odds ratio between 2 and 3 for anxiety, sleeping problems and severe pain after adjustment for age, education, marital status, and employment)
Taloyan, M Wajngot, A.; Johansson, S. E.; Tovi, J.; Sundquist, J.	Poor self-rated health in adult patients with type 2 diabetes in the town of Sodertalje: A cross-sectional study	To investigate whether there was an association between ethnicity and poor self-rated health in subjects with type 2 diabetes and to analyze whether the association remained after adjusting for possible confounders	2010	A cross-sectional survey based on a patient population in the town of Södertälje.	Unconditional logistic regression was performed to estimate the odds ratios (ORs) and 95% confidence intervals (95% CIs). Interviews. questions the same as in the Swedish Level-of-Living Surveys (SALLS), expanded with questions pertinent for immigrants (e.g. migration back-ground, knowledge of Swedish).	A total of 354 individuals were included: Assyrian/Syrian-born (n = 173) and Swedish-born (n = 181). mean age 37.5 years Age was categorized into three groups: 32–59, 60–69 and ≥70 years	Odds ratio for rating poor SRH for Assyrian/Syrian subjects with type 2 diabetes was 4.5 times higher (95% CI = 2.7–7.5) than for Swedish patients in a crude model. After adjusting for possible confounders, unemployed/retired people had 5.4 times higher odds for reporting poor SRH than employees (OR = 5.4; 95% CI = 2.3–12.5). Women had 1.8 times higher odds (95% CI = 1.0–3.0) for reporting poor SRH than men. Unexpectedly, the youngest age group (32–59) had poorer health than the older age groups

**Table 5.** Quality Evaluation of the Quantitative and Qualitative Studies.

**a) Studies included**						
Study ID	Country	Sample size	Age	Qual	Quant non RCT	Quant descriptives
Abuelmagd W, Hakonsen H, Mahmood K Q, Taghizade N, Toverud EL.	Norway	120	29–80		x	
Diaz E, Kumar BN, Gimeno-Feliu LA, Calderon LarranagaA, Poblador-Pou B, Prados-Torres A.	Norway	67,398 refugees, 66,942 labour immigrants, 101,276 family reunification immigrants and 16,379 education immigrants,	29–36			x
Emami A, Tishelman C	Sweden	45	25–70	x		
Emami A, Ekman S.	Sweden	30	>75 år	x		
Kessing, L L, Norredam M, Kvernrod AB, Mygind A, Kristiansen M.	Denmark	29	50–69	x		
Koochek, A, Johansson, S. E, Kocturk, T. O, Sundquist, J. Sundquist, K.	Sweden	402	60–84			x
Koocheck A, Montazeri A, Johansson SE, Sundquist J	Sweden	298 + 176 + 151 = 626	60–84			x
Kristiansen M, Lue-Kessing L, Mygind, A, Razum O, Norredam M.	Denmark	29	50–69	x		
Kristiansen M, Thorsted BL, Krasnik A, Von Euler-Chelpin M.	Denmark	84,489				x
Le, M., Hofvind S, Tsuruda K, Braaten T, Bhargava S.	Norway	885,979	50–69		x	
Lindstrom M, Sundquist J	Sweden	5600	18–65		x	
Molsa, M, Punamaki RL, Saarni SI, Tiilikainen M, Kuittinen S, Honkasalo ML.	Finland	128 + 128	50–80		x	
Steiner KH, Johansson SE, Sundquist J, Wandell PE.	Sweden	526 + 2854 (S)	27–60		x	
Taloyan M, Wajngot A, Johansson S, Tovi, J, Sundquist J.	Sweden	354	60–69		x	

**b) Quantitative papers - non RCT**						
Study ID	Quant non RCT	Are the participants respresentative Of the target population?	Are measurement appropriate regarding both the outcome and the intervention?	Are there complete outcome data?	Are the confounders accounted for in the design and analysis?	During the study period is the intervention administered (or exposure occurred) as intended?
Abuelmagd W, Hakonsen H, Mahmood K Q, Taghizade N,Toverud EL.	cohort	yes	Yes (convenient invited)	yes	yes	yes
Koocheck A, Montazeri A, Johansson SE, Sundquist J	Cross sectional	yes	yes	yes	yes	yes
Le, M., Hofvind S, Tsuruda K, Braaten T, Bhargava S.	Cohort	yes	yes	yes	yes	yes
Lindstrom M, Sundquist J	Cross sectional	yes	yes	no	yes	yes
Molsa, M, Punamaki RL, Saarni SI, Tiilikainen M, Kuittinen S, Honkasalo ML.	Cohort	yes	yes	yes	yes	yes
Steiner KH, Johansson SE, Sundquist J, Wandell PE.	Cross sectional	yes	yes	yes	yes	yes
Taloyan M, Wajngot A, Johansson S, Tovi, J, Sundquist J.	Register	yes	yes	yes	yes	yes

**c) Quantitative descriptive papers**						
Study ID	Quant descriptive	Is the sampling strategy relevant to address the research question?	Is the sample representative of the target population?	Are the measurements appropriate?	Is the risk of nonresponse bias low?	Is the statistical analysis appropriate to answer the research question?
Diaz E, Kumar BN, Gimeno-Feliu LA, Calderon LarranagaA, Poblador-Pou B, Prados-Torres A.	Invited register- questionnaire	yes	yes	yes	yes	yes
Koochek, A, Johansson, S. E, Kocturk, T. O, Sundquist, J. Sundquist, K.	Cross sectional	yes	yes	yes	yes	yes
Kristiansen M, Thorsted BL, Krasnik A, Von Euler-Chelpin M.	Register retro, invited /participated or not	yes	yes	yes	no	yes

**d) Qualitative papers**						
Study ID	Qual	Is the qualitative approach appropriate to answer the research question?	Are the qualitative data collection methods adequate to address the research question?	Are the findings adequately derived from the data?	Is the interpretation of results sufficiently substantiated by data?	Is there coherence between qualitative data sources, collection, analysis and interpretation?
Emami A, Tishelman C	Focus groups int	Yes	Yes	Yes	Yes	Yes
Emami A, Ekman S.	In depth interview	Yes	Yes	Yes	Yes	Yes
Kessing, L L, Norredam M, Kvernrod AB, Mygind A, Kristiansen M.	Semistructured interviews	Yes	Yes	Yes	Yes	Yes
Kristiansen M, Lue-Kessing L, Mygind,A, Razum O, Norredam M.	Semistructured interview	Yes	Yes	Yes	Yes	Yes

The studies’ approaches varied from focusing specifically on older immigrant women to including partial information about them in larger samples. The quantitative studies more often included older immigrant women as a subgroup in a wider sample of the general population, allowing comparisons with other age groups, gender, and country backgrounds. Only three of the quantitative studies (Abuelmagd et al., 2018; Kristiansen et al., 2012; Le et al., 2019) used a sample consisting of only female respondents, and only one (Abuelmagd et al., 2018) included exclusively older (mean age 55.7 years) immigrant women as the respondent group. In addition, although two of the quantitative studies (Abuelmagd et al., 2018; Diaz et al., 2015) used a sample consisting exclusively of a migrant population, the others used the host countries’ population as a reference. However, all the qualitative studies exclusively described and analyzed the immigrant population by focusing on either a specific country background or several nationality backgrounds subsumed under broader terms such as “immigrant population” or “ethnic minorities”. Four of these five studies focused exclusively on female informants. In accordance with the aim of this study, healthcare aspects of socioeconomic disparity were identified in all the studies: cancer, diabetes, multimorbidity, pain/anxiety/sleeping problems, physical activity, mental health, quality of life, healthcare services, and gerontology.

### Poorer Health among Older Women Compared to the Majority Population and Men

The quantitative studies in this review examined the social or economic implications for health by comparing immigrant women's health behavior with either the majority population in the host country or men. The studies covered different healthcare issues and drew attention to social or economic variables to varying degrees, often focusing on the largest immigrant groups in the respective Nordic countries and using the majority population as a reference group. This is reflected, for example, in the differences between immigrants’ self-reported disease burdens. In a study of Turkish-born immigrants, using a Swedish Survey of

Living Conditions, Steiner et al. (2007) found a significant difference in self-reported health (SRH) compared to the general Swedish population. Older age among Turkish women was associated with an increased risk of severe pain, anxiety, sleeping problems, lower education, and unemployment. Turkish-born men also showed a higher risk of anxiety, sleeping problems, and severe pain compared to the Swedish controls, but to a lesser degree than the Turkish women (Steiner et al., 2007).

Similarly, another Swedish study showed that Assyrian/Syrian-born individuals reported poorer health than the general Swedish population (Taloyan et al., 2010). Taloyan et al. (2010) found that Assyrian/Syrian respondents with type 2 diabetes had significantly higher odds of reporting poorer SRH than Swedish-born respondents, and the odds were highest for Assyrian/Syrian women. The study also showed that unemployment and retirement were significantly related to poor SRH and were higher for the immigrant group. Although the study reported that the older age groups (60–69 years) suffered from poor health, the authors surprisingly also found that the youngest age group (32–59) had even poorer health (Taloyan et al., 2010). Similarly, a questionnaire-based study, Abuelmagd et al. (2018) found that one-third of 120 Pakistani women with type 2 diabetes in Oslo reported poor health. A higher proportion had less than 10 years of education, and the majority reported that they needed assistance to understand medical information written in Norwegian. The study sample included two-thirds of the women in the age range of 51–80 years (Abuelmagd et al., 2018). A comparison of health status in a Finnish study between Finnish-born individuals (N = 128) and Somali refugees (N = 128) ranging in age from 50 to 80 years indicated a lower self-reported health status and quality of life among the Somali respondents (Molsa et al., 2014). The study reports that anxiety/depression levels were considerably higher among older Somalis than among Finns, and that Somali women reported far worse mental health than their male counterparts. The authors found no gender differences in the Finnish group (Molsa et al., 2014). A Swedish study of older Iranian-born immigrants (Koochek et al., 2007) reported similar findings. Iranians reported poorer health-related quality of life (HRQL) than Swedes. The HRQL of Iranians did not decrease with length of residence in Sweden; it increased for Iranian women but not for men (Koochek et al., 2007). Length of residence and multimorbidity among immigrants were also considered in Diaz et al.’s (2015) Norwegian study. The authors found that multimorbidity was significantly higher among refugees upon arrival to Norway, but multimorbidity also increased rapidly in particular for female labor immigrants. For labor immigrants outside Europe and North America, multimorbidity was more associated with old age and women. A cautionary note here is that although the sample consisted of age groups from 15 to older than 65, those younger than 44 made up more than 80% of the sample population (Diaz et al., 2015).

Higher prevalence of risk factors for cardiovascular disease in immigrants, combined with the increasing important explanatory socioeconomic differences in health, has prompted studies about immigrant groups’ sedentary lifestyle behavior and physical activity (Koochek et al., 2008; Lindstrom & Sundquist, 2001). In a population-based Swedish study, Koochek et al. (2008) showed that older Iranian-born women had significantly higher body mass index (BMI). The authors found no significant differences in BMI between Swedish men (reference group) and Swedish women or Iranian men. Lindstrom and Sundquist (2001) also found that immigrant women, including those from Arabic-speaking countries and other non-European countries, had significantly higher odds of having sedentary leisure time than women and men born in Sweden (Lindstrom & Sundquist, 2001).

### Lower Attendance to Health Screening Programs among Older Immigrant Women

The Nordic countries offer organized mammography screening, usually with a high turnout. Therefore, the trend of lower turnout among immigrants has been a focus in studies of immigrant women (Kristiansen et al., 2012; Le et al., 2019). Kristiansen et al. (2012) showed in a large cohort study (N = 84,489) that older (60–64 years) non-Western women are least likely to participate in the Danish mammography screening program. In Le et al.'s study (2019), participation rates for breast cancer screening in Norway were lowest among immigrants across sociodemographic factors, such as lower income, unemployment, and less than 10 years of education. Compared to Norwegian-born women, being from other parts of Western Europe, Eastern Africa, and Asia was significantly associated with lower participation, but participation increased with longer residence in Norway. More than 33% of the study sample consisted of women in the 55–69 age group (Le et al., 2019).

### Experiences of Inequality in Health among Immigrant Women

The qualitative studies in this review explored the experiences of social inequality among immigrant women in their everyday live. Low literacy, lower income, or cultural beliefs were prominent characteristics of the immigrant women's experiences of health and well-being. In the Danish study that explored older immigrant women's experiences of participation in mammography screening, for example, Kristiansen et al. (2014) described how the women felt about breast cancer screening. The women perceived the risks of breast cancer as no worse than those for other types of cancer, diabetes, infectious diseases, cardiovascular diseases, or mental health problems, which were also common in their communities. The women also wondered why screening programs targeting those diseases were lacking: “I recall when we came to Denmark, then we were checked for a lot of stuff [diseases], but now we only get invited for the breast, but besides that we don't get asked [invited for] any other investigation (Somali, group interview)” (p. 210).

The focus groups, consisting of immigrants from Somalia, Turkey, Pakistan, and Arab countries, had lower rates of educational attainment and employment, and only a few had attended mammography screening programs (Kristiansen et al., 2014). Similarly, in contextualizing immigrants’ health behavior, another interview study (Kessing et al., 2013) of 29 older immigrants (from Somalia, Turkey, India, Iran, Pakistan, and Arabic-speaking countries) living in Sweden emphasized the implications of women's transnational obligations and competing everyday priorities for participation in mammography screening. Struggles in securing financial help for relatives abroad and problems in their everyday lives left little time for self-care and mammography screening. Trying to establish “a social life in Denmark while helping relatives in their country of birth who suffered from ongoing war and poverty” was more pressing (Kessing et al., 2013; pp. 7–8). Their transnational family obligations often made a direct impact on the everyday life in their host country. For example, one participant described her concerns about a sick mother living alone in India:

“My mom she is almost 80, 80 plus she is, but she lives all alone, all alone (…) I call her every day: “Mom, are you alright? Did you take your medicines?” (…) Every day I can only think about what about in the evenings, if she is still alive, if she needs some help.” (p. 7)

Older women's everyday life struggles as a contribution to disparity in somatic and mental health experiences were often due to their immigrant background. Emami and Ekman’s (1998) study of older Iranian immigrants in Sweden showed how living in a new country affected their perceptions of health and well-being. The women experienced difficulties establishing social relations without speaking the host country's language and had no friends of their own age. Limited activities because of language barriers resulted in a narrower social network consisting mainly of relatives. However, some, despite being materially well off, lacked active social lives:

“I cannot complain of anything here; I have almost everything I ever need, but nothing is the same as ever. Every day, I used to be visited by relatives, friends, and neighbors in Iran as well. Here, nobody knocks at my door.” (Mrs. Tooran, a 75-year-old widow, p. 191)

Although the participants described themselves as healthy, they complained of diffuse pain, abdominal pain, headaches, and sleeplessness. Some also gave the impression of not having specific plans for their future and trusting in fate (Emami & Ekman, 1998).

Differing ideals of health and illness also seem to affect health help-seeking behavior of immigrant women (Emami & Tishelman, 2004). Despite experiences of reduced well-being because of a suboptimal social life, lack of a sense of meaning and belonging, and physical disorders, many older women in Emami and Tishelman’s (2004) study stated that a positive attitude would cure their illnesses. Some of the older women, for example, questioned the “negative” view of menopause and the post-menopausal hormone replacement treatment prescribed by physicians. A 50-year-old housewife explained:

“I don't understand all this negative talk about menopause (in Sweden). Our mothers haven't had any menopausal problems. I think it is healthy. This is a part of preparing to become old. A sort of retreat. My woman's body has done its job. Now is time for retreat. Menopause helps me to understand this aging phase and be better prepared for it.” (p. 82)

The women had a positive life-cycle view of menopause, something which might affect their health-seeking behavior and thus reinforce health inequalities compared to the majority population.

## Discussion

The review studies were examined and integrated to shed light on factors associated with health inequalities, and the results highlight differences in social inequalities in health between the majority population and men as well as experiences of inequality in health among immigrant women. Older women born in Asia, Africa, and South America living in the Nordic countries seem to fare worse in many health indicators compared to immigrant men and the majority population at large. These differences were emphasized in many of the studies related to various health issues, such as anxiety, depression, diabetes, multimorbidity, sedentary lifestyle, and quality of life. Lower participation in cancer screening programs is also a distinctive feature among immigrant women, which could be related to the immigrant women's help-seeking behavior. Transnational family obligations and local responsibilities leave little room for prioritizing self-care, but differing views of health conditions might also contribute to avoidance of healthcare services.

Overall, the studies were of good quality, but they included the target population of older immigrant women to varying degrees. In many of the studies, especially those with a quantitative design, to the extent that they included people with immigrant backgrounds, it was for comparison with the majority population and not to develop knowledge of older immigrant women's specific challenges and resources. Although we used a relatively low age limit (50 years) for “older” women, only a few of the studies paid specific attention to older women. The qualitative studies targeted older immigrant women more specifically and explored individual ethnic group experiences and “migrants” as a group. Although some of the studies clearly showed a link between social inequality and health, others were more comprehensive and revealed what seems to be an indirect impact of social inequalities on health status.

Many studies have drawn attention to the association of health inequalities with socioeconomic factors globally. The studies showed that older people are more at risk of illness and health problems (Beard et al., 2016), but there are also, as our review shows, gender differences. Studies outside the Nordic countries have long emphasized how social inequalities disadvantage women health (Charpentier & Quéniart, 2017; Mackenbach et al., 2008). Older women are more likely to experience general health problems, including work-related stress, discrimination, and physical hazards (Payne & Doyal, 2010). Gender, social status, and race might, therefore, in combination explain adverse effects on health status (Rosenfield, 2012). In agreement with our review of studies conducted in the Nordic countries, older immigrant women are worse off when it comes to health status considering their social position (Conkova & Lindenberg, 2019; Jetten et al., 2018). Scholars have suggested that social disadvantage is higher among older women with a non-White ethnic background, and that this disadvantage contributes to a heightened risk of adverse health outcomes (Anand et al., 2006). The combination of disadvantaged social positioning and negative health outcomes has sparked a discussion of older immigrant women's risks conceptualized as multiple jeopardy. The accumulation of negative effects due to people's stigmatized statuses, such as being female, older, immigrant, and having lower socioeconomic status (Chappell & Havens, 1980), is precisely what our review suggests might contribute to increased illness and health problems among immigrant women in the Nordic countries.

Differing from our findings, other studies have previously shown a relative health advantage and even higher life expectancy in favor of immigrants in general, testifying to a “healthy immigrant effect” (Biddle et al., 2007; Mehta et al., 2016; Vang et al., 2017). Nevertheless, these advantages often seem to fade away with acculturation and socioeconomic deprivation in later life (Biddle et al., 2007; Hollander et al., 2020; Mehta et al., 2016). There is also an indication that immigrants’ health advantages are more salient regarding mortality rates than morbidity (Vang et al., 2017). Furthermore, in a study of life expectancy in six European countries researchers found that mortality patterns across the migrant populations were heterogeneous and varied across sex, age group and country of destination (Ikram et al., 2016).

Despite heightened awareness and efforts in the last few decades to reduce the impact of social inequalities on health in Europe, including the Nordic countries, inequalities have persisted (Kravdal, 2013; Mackenbach et al., 2008, 2016). Furthermore, as this review showed, few studies have explored the issue of social inequalities and healthcare with a focus on immigrant populations in the Nordic countries and even fewer considered older immigrant women. This may have to do with the notion that the Nordic countries’ universal welfare system grants free healthcare to everyone. Studies also suggest that including minorities is considered a costly and time-consuming process (Morville & Erlandsson, 2016). In addition, the meager record of including minorities in research seems to be due to researchers’ insufficient education and training, as well as motivation and incentives, in designing and implementing population-based studies of minorities (Burchard et al., 2015).

Excluding immigrants from research is ethically and scientifically unacceptable (Morville & Erlandsson, 2016). The research portfolio of studies specifically designed for multicultural society therefore needs to be expanded. This absence of research-based evidence poses a major barrier to developing effective integration and health-promoting strategies for immigrant populations in the Nordic countries. Including older people in research requires considering accessible communication possibilities, building relationships, and having flexible approaches (Schilling & Gerhardus, 2017). Moreover, ensuring grant applications focusing on minorities, developing the careers of minority scientists, and facilitating and valuing research on minorities by investigators of all backgrounds might contribute to the development of relevant research-based evidence (Burchard et al., 2015).

### Limitations and Strengths

Although the results of these studies should be considered when planning policy measures targeting older immigrant women, the implications should be assessed with some of the limitations of the studies in mind. This review assessed only studies from the Nordic countries. Immigrant women's experiences within the frame of these countries’ universal welfare systems might not be transferable to other countries, such as those with insurance-based healthcare systems. The review focused only on immigrants from Asia, Africa, and South America, and their experiences might not transfer to other immigrant groups’ health-seeking behavior. This review included primary studies in English and Scandinavian languages from major databases, which is a strength of this study.

### Implications for Practice

This review has uncovered the need for large sample studies of women within specific immigrant groups and immigrants in general. Although comparisons with the majority population are necessary, there is a need to include diverse minority populations to consider differing sociodemographic factors among various groups of migrants (Le et al., 2019). This is even more important considering the modest number of older immigrants in the Nordic countries; as a subgroup sample in larger studies, older immigrants might often be too few. In addition, this review uncovered a lack of studies focusing on social inequalities and how they are experienced in immigrant women's everyday lives to get a better understanding of women's health behavior (Kessing et al., 2013). There is also a need to tap into immigrant women's experiences before and after migration to a new country, focusing specifically on their immigration status as refugees or working migrants, and how this affects their health-seeking behavior (Kristiansen et al., 2014). To uphold nurses’ obligation to ensure fairness and social equity, they need to be aware of older immigrant women's possible economic and health status disadvantages and reduced access to healthcare services.

## Conclusions

The few studies found in this review indicate that older immigrant women in the Nordic countries have poorer health and less access to healthcare services than the majority population and men in general. This review demonstrated that healthcare services and health status for immigrant women seem to be connected to their socioeconomic status, contributing to positioning them in a multiple jeopardy situation. Consequently, this may endanger nurses’ professional obligation to adhere to fairness and social equity in healthcare.
